# Single-Centre Experience of Gallbladder Cancer in the United Kingdom: Do Patients With Incidentally Discovered Malignancy After Gallbladder Surgery Have Better Outcomes?

**DOI:** 10.7759/cureus.107719

**Published:** 2026-04-25

**Authors:** George Eskandar, Mohamed Abuahmed, Rahel Rashid, Ahmed Hammouda, Jeremy Wilson, Conor Magee

**Affiliations:** 1 Upper Gastrointestinal Surgery, NHS University Hospitals of Liverpool Group, Liverpool, GBR; 2 Vascular Surgery, Sheffield Teaching Hospitals, Sheffield, GBR; 3 General Surgery, NHS University Hospitals of Liverpool Group, Liverpool, GBR; 4 General Surgery, Wirral University Teaching Hospital NHS Foundation Trust, Wirral, GBR

**Keywords:** biliary tract cancer, gallbladder cancers, gallbladder surgery, liver resection, lymphadenectomy, palliation in surgery

## Abstract

Background

Biliary tract cancers refer to a spectrum of invasive tumours, usually adenocarcinomas, arising from the gallbladder or cystic duct (gallbladder carcinoma or GBC) or the biliary tree (cholangiocarcinoma or CCA) and represent a minority of all human cancers. Risk factors of GBC are old age, female gender, and gallstones, which represent the strongest risk factor, in addition to congenital biliary tree malformations and obesity.

Methods

This was a retrospective review using electronic records of all the patients admitted at a single centre in the UK, between January 2014 and July 2024, who were diagnosed with GBC or dysplasia. The study included 47 patients.

Results

Forty-seven patients were analysed, with a median age of 72 years. Forty-one of them were female patients, and six were male patients, with a mean BMI of 25.5 and the mode of American Society of Anesthesiologists (ASA) was three. Approximately 80% of the patients had gallstones, and about 70.2% of the cases were diagnosed before having an operation, if any. More than half of the patients (55.3%) had metastatic advanced cancer at the time of diagnosis. Moreover, about 81% of the patients were managed palliatively with either palliative chemotherapy or best supportive care. However, overall mortality was high (76.5%).

Conclusion

The management of gallbladder cancer requires a multidisciplinary approach, with surgery, chemotherapy, and radiotherapy playing pivotal roles. Our study showed that incidentally discovered gallbladder cancer carries better survival outcomes than symptomatically evident cancer. Radical surgery combined with adjuvant therapies offered the best survival outcomes for early-stages (TIb, T2) and locally advanced GBC (T3). Further research is needed to explore different modalities to screen, diagnose early, and effectively treat advanced cases of GBC, with better survival results.

## Introduction

Gallbladder cancer (GBC) is the most prevalent form of biliary tract cancer and ranks sixth among the most common gastrointestinal malignancies worldwide [1,2]. It is characterized by poor prognosis, high recurrence rate, and a five-year survival rate of only 10%, as most of them are diagnosed at an advanced stage [3]. GBC has a female predominance with a female patient-to-male patient ratio of 5:1, which is attributed to the higher incidence of gallstone disease and presence of the female hormone oestrogen, and is most prevalent among the elderly, with a mean incidence in the seventh decade [4].

Gallstones represent the most important risk factor; 85% of GBC patients have gallstones [5], especially if the gallstones are larger than 3 cm or are cholesterol stones [6]. The pathophysiology behind this is the chronic irritation caused by gallstones, which leads to dysplasia and, consequently, adenocarcinoma formation. However, only 1% of gallstones develop GBC [7]. Other risk factors include gallbladder calcifications, most importantly, porcelain gallbladder. A recent review of a large cohort of 60,781 patients who underwent cholecystectomies found that the incidence of GBC was 6% among patients with porcelain gallbladder [8]. In addition, family history, obesity, smoking, sclerosing cholangitis, and infection with Salmonella typhi and Helicobacter species represent less common risk factors for GBC [9].

The majority of patients with GBC often present incidentally during routine cholecystectomy for gallstone disease (60%) [4]. It is detected in 0.2-1.1% of patients who undergo laparoscopic cholecystectomy, and represents 27-72% of all newly diagnosed gallbladder tumours. Following laparoscopic cholecystectomy and histopathological analysis, staging CT is necessary, as up to 40% of patients with incidental GBC have residual disease, most of which is in the gallbladder fossa and lymph nodes. Diagnostic workup helps to assess the probability of residual cancer in the cholecystectomy bed or regional lymph nodes and to diagnose distant metastasis. These are incidentally discovered [10] at an advanced stage with symptoms such as right upper quadrant or epigastric pain, jaundice, nausea and vomiting, anorexia, and weight loss (40%) [11].

GBC is staged according to the depth of tumour invasion (T), presence and number of lymph node metastases (N), and presence of distant metastases (M), based on the eighth edition of the Union for International Cancer Control (UICC) [4]. Workup for GBC includes blood tests: full blood count (FBC), liver function tests (LFTs), and tumour markers including Carcinoembryonic Antigen (CEA) and Cancer Antigen (CA) 19-9 [12]. This is followed by imaging, which includes: Ultrasound scan (USS), which is the primary modality by which the gallbladder is assessed, and lesions like gallstones or GBC can be detected. However, the greyscale and operator dependence act as challenges to the ability of USS to detect GBC. Evaluation of the depth of invasion appears better in new ultrasonography techniques such as endoscopic ultrasonography (EUS) and high-resolution ultrasonography (HRUS) [13].

CT scan represents the mainstay investigation for diagnosis and staging of GBC, with a diagnostic accuracy for the assessment of T-stage of about 85%, with 100% sensitivity for discrimination of T4 lesions, and 79% for the differentiation between T1 and T2 lesions. However, over-staging can be caused by duodenal infiltration on CT, which might not be evident during surgery, while under-staging can be caused by the low sensitivity of CT for peritoneal (30%) and distant lymph node (LN) metastases (20%) [14]. MRI with gadolinium-enhanced contrast, on the other hand, has the advantage of differentiating between chronic cholecystitis and GBC wall thickening, which is one of the major drawbacks of other imaging modalities. Although both CT and MRI have a low sensitivity for distant LN and peritoneal metastases, positron-emission tomography (PET) detects high glucose uptake of tumour cells, with a sensitivity to detect distant and LN metastases is 85-100% and 67-71%, respectively [15].

Although histopathological diagnosis is not needed to confirm the diagnosis, especially if the GBC is confirmed by imaging to be operable, biopsy is required to confirm the histological subtype of the cancer in non-operable and/or metastatic lesions to guide chemotherapy. Biopsies could be done either percutaneously or by Endoscopic Retrograde Cholangiopancreatography (ERCP) in case of associated biliary obstruction, for which ERCP will be both diagnostic and therapeutic [16]. Finally, staging laparoscopy has a greater role in locally advanced (T3/T4) than in early cancer stages, where its role is to diagnose disseminated disease and/or inoperable cases, which were not detected by cross-sectional imaging [17].

The management of GBC involves a multidisciplinary approach (MDT), and it includes: surgery, adjuvant therapies, and palliative care. Surgical resection remains the cornerstone of treatment, where radical cholecystectomy with R0 resection and appropriate lymphadenectomy is the standard surgical approach, offering the best chance for long-term survival [18]. However, due to the advanced stage at diagnosis in many cases, adjuvant therapies, such as chemotherapy using cisplatin/gemcitabine which are used in advanced, unresectable case, are crucial for improving outcomes. Capecitabine has emerged as an option for adjuvant chemotherapy following a phase III study [19], while radiotherapy is primarily used in the adjuvant setting, with ongoing research into its role in neoadjuvant and palliative contexts [20]. Finally, for patients with unresectable or metastatic GBC, palliative care focuses on symptom management and improving quality of life, i.e. best supportive care (BSC), in addition to systemic therapy combinations, which have shown some success in managing advanced disease, although the prognosis remains poor [21]. This study aimed to review the management of GBC in one of the high-volume centres in the UK based on the recent guidelines and the effect of this management on patients’ survival. 

## Materials and methods

This was a retrospective cohort study conducted from January 2014 to July 2024, in which 1521 patients who were admitted for gallbladder disease were identified and screened using retrospective electronic data records at the Wirral University Teaching Hospital NHS Trust (WUTH) in the UK. Forty-six patients, aged ≥18 years, with histologically proven GBC were included in this study. Patients with incomplete records, diagnosed with gallbladder polyps, benign gallbladder disease, or cholangiocarcinoma were excluded from this study. Patients' demographic data, operation type, presence or absence of gallstones, surgical morbidity and mortality, timing of diagnosis, histopathological classification, cancer stage, and survival data were collected for further analyses. Overall survival was calculated from the time of diagnosis until the time of death or the last time of follow-up, which was in July 2024.

The distinction between simple cholecystectomy and radical resection is critical in the management of GBC. Simple cholecystectomy involves the removal of the gallbladder alone, while radical resection includes the former procedure along with extensive lymphadenectomy surrounding the liver hilum, the cystic duct, the hepato-duodenal ligament, the celiac trunk, and retro-duodenal tissue with wedge resection of hepatic segment IVb, which represents the gallbladder bed [22]. Patients with unresectable disease were offered palliative therapies, including chemotherapy using cisplatin/gemcitabine. The primary outcome was survival following treatment, including disease-free survival.

Survival analyses were performed using the Kaplan-Meier method. Differences in survival rates were analysed by the log-rank test. Normality testing, i.e., the Shapiro-Wilk test, was used to assess normal distribution. A Student's t-test and ANOVA test were used to analyse quantitative variables if normally distributed, or the Mann-Whitney test if not normally distributed. A chi-square test and Fisher's Exact test were used to determine differences in frequencies. 

For all calculations, Jamovi (version 2.6) (Computer Software). Retrieved from https://www.jamovi.org, an open-source statistical software was used. A p-value of less than 0.05 was considered statistically significant. This study was approved by the clinical governance committee at the Wirral University Teaching Hospital for a retrospective study (reference no Sur242547).

## Results

Patients and surgery

Forty-six patients were included in this study. Of these, 40 (86.9%) were female subjects, and six (13%) were male subjects. The median age was 72 years (range 47-93 years). In addition, there were no significant differences between the two groups (72.7 ± 11.6 years for female patients, 69 ± 9.3 years for male patients, *p*=0.46). Mean BMI was 25.5 (±5), the mode of the American Society of Anesthesiologists (ASA) was three, and the median follow-up was 57 months (interquartile range or IQR = 118 months).

The number of patients diagnosed with GBC showed a significant discrepancy throughout all the years of data collection in this study, wherein the effect of COVID-19 was demonstrated by zero cases diagnosed in 2020, compared to eight cases in 2019 and seven cases in 2021.

Patients were then divided into two groups: the first was the incidental group, who had their GBC diagnosed incidentally after laparoscopic cholecystectomy, and the second was the symptomatic group, which included patients who presented with symptoms of GBC and were diagnosed by cancer workup investigations.

Incidental Group Following Laparoscopic Cholecystectomy

This group included 12 patients with an incidental finding following surgery for gallstones, by histopathological analysis. Among all 12 patients, a preoperative USS was done in 11 patients (91.6%), six (54.5%) patients had an ultrasound finding of a thick-walled gallbladder, while two patients (18.1%) had polypoid lesions detected by USS, and confirmed to be adenomatous polyps by histopathology. Regarding other imaging modalities, computed tomography of abdomen and pelvis (CT AP) with intravenous (IV) contrast was performed in seven patients (58.3%), magnetic retrograde cholangiopancreatography (MRCP) was done in four patients (33.3%) and EUS +/- ERCP was done in two patients (16.6%). Table 1 shows the indications and findings of each imaging modality among patients.

**Table 1:** Findings of various imaging modalities among patients who underwent surgery for gallstones and had an incidental finding of GBC postoperatively GBC: gallbladder cancer; CT AP: computed tomography of the abdomen and pelvis; USS: ultrasound scan; MRCP: magnetic retrograde cholangiopancreatography; CBD: common bile duct; LFTs: liver function tests; EUS: endoscopic ultrasound; ERCP: endoscopic retrograde cholangiopancreatography.

Imaging modality	Indication	Findings
CT AP	Abdominal pain and fever	Gallstones and sludge within the otherwise normal gallbladder
	Abdominal pain	Multiple gallstones in the gallbladder
	Node-positive left breast cancer staging CT	Tiny gallstone in the gallbladder + prominent mesenteric nodes, the largest measuring 16 mm
	A 20 mm polyp in the gallbladder was detected on USS.	Stable wall confined 21 mm dominant gallbladder polyp. No radiological evidence of malignancy
	Further investigation of gall bladder adenomyomatosis on USS	No abnormal thickening of the gallbladder wall in keeping with adenomyomatosis
	Abdominal pain	Thin-walled gallbladder
	Abdominal pain	Marked calculous cholecystitis and the pericholecystic collection
MRCP	Dilated CBD on USS	Gallstones in the gallbladder with mild biliary dilatation
	Contracted thick-walled gallbladder for surgery	generalised adenomyomatosis
	Obstructive jaundice, gallstones	No gallbladder wall thickening, no CBD stones
	Suspicious appearance of the gallbladder on USS	perforated calculous cholecystitis, resulting in pericholecystic fluid, infective rather than neoplastic
EUS +/- ERCP	Raised LFTs	Thick-walled gallbladder with stones and sludge, no CBD stones
	Gallbladder and CBD stones detected by USS and MRCP	CBD stones, extracted

Intraoperatively, the laparoscopic cholecystectomy of three patients was converted to open surgery. Postoperative histopathological analysis revealed that seven patients (58.3%) had a localised tumour (i.e., T1/T2), without nodal or distant metastasis. Regarding further management for those seven patients, laparoscopic cholecystectomy was sufficient in four patients (33.33%) who had T1a tumour, while re-resection was performed in three patients (25%) who had T1b tumour, in the form of liver resection with lymphadenectomy.

On the other hand, the pathological analysis revealed that four patients (41.6%) had advanced malignancy, two of them (16.6%) had T3 cancers and were treated by further radical resection in the form of liver resection and lymphadenectomy. The other two (16.6%) had stage IV malignancy, the first of which was further treated by palliative chemotherapy (cisplatin/gemcitabine/durvalumab), while the second was further treated by bile duct resection, liver resection, omental resection, and hepatico-jejunostomy, followed by the creation of a diversion ileostomy for peritoneal metastasis. 

The postoperative complications following surgery in this group included two patients had postoperative obstructive jaundice, which required ERCP plus stent insertion (Clavien-Dindo III). Finally, none of the patients died from postoperative complications.

Symptomatic Group

This group included 34 patients. In 33 patients (70.2% of the total cohort), the diagnosis of GBC was made without surgery. Diagnosis was suspected by manifesting symptoms of GBC, which indicates a late stage. In one patient, however, GBC was suspected intraoperatively during the formation of a diversion stoma for large bowel obstruction (LBO), which warranted further investigations, leading to the diagnosis of GBC.

Among the 34 patients, 27 patients had a stage IV tumour at diagnosis, while seven patients had a stage III tumour at diagnosis. None of the patients of this group were diagnosed at an earlier stage (stage I/II).

Overall survival

The estimated one-year survival rate was 34%; while the three-year survival rate was 8.6%. The five-year survival rate was 6.3%, and finally, the 10-year survival rate was 2.1%. There was an obvious, however, not statistically significant difference in survival between male subjects and female subjects (11.6 ± 12.4 versus 13.7 ± 23.9 months, *p*=0.85). Concerning age differences, 18 patients (38%) were younger than 70 years. Again, there was no statistically significant difference in survival rates of patients younger than 70 years compared to those older than 70 (19.3 ± 28.7 versus 9.8 ± 17.5 months, *p*=0.16).

**Figure 1:**
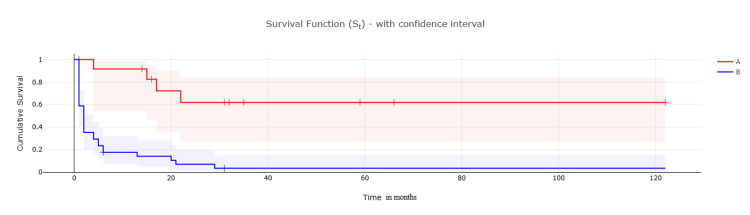
A comparison between the surgery group (A) and palliative group (B) in terms of survival rates (in months) Kaplan-Meier Curve with time in months.

Regarding the difference in survival between both study groups, we analysed that the median overall survival (MOS) was not reached in patients with incidental GBC (surgery group), which means that more than 50% of the patients are still alive, whereas patients with suspected preoperative GBC (palliative group) displayed a significantly shorter MOS of two months (χ²=20.3104, *p*<0.05). When performing the log-rank test, there was a significant difference in mortality between the two groups (χ²=15.008). This can be demonstrated by Figure 1, which shows Kaplan-Meier curve plot comparing both groups in terms of survival.

Union for International Cancer Control (UICC) staging

According to the eighth edition of the UICC, GBC stages range from stage 0 to stage IVB. In this study, two of the 46 patients (4.34%) had stage I disease (T1N0M0), five patients (10.8%) had stage II disease (T2N0M0), and 11 patients (23.9%) had stage III disease (T3N0M0/T1-2-3N1M0). Finally, 26 patients (56.4%) were diagnosed with stage IV. Two patients had an unknown stage.

Stage IV vs Stages I, II, and III

Twenty-six patients (56.5%) presented with metastases (stage IV) at the time of primary diagnosis; one of these patients was diagnosed after surgery for gallstones and another patient was treated by diversion ileostomy for associated LBO and peritoneal metastasis together with palliative chemotherapy in the form of cisplatin/gemcitabine, while durvalumab was added to the regimen in the former patient. The rest of 26 patients with stage IV tumours were only treated by either: palliative chemotherapy using cisplatin/gemcitabine regimen (five patients, 19.2%), Folfox (a combination of folinic acid, fluorouracil, and oxaliplatin) was given following the former regimen in another patient, while carboplatin was the lone chemotherapeutic agent in another patient. Finally, BSC was the only treatment for the remaining 17 patients (65.5%). Mortality in stage IV patients was 25 out of 26 patients (96.1%).

Stage III, on the other hand, included 11 patients; two patients were treated surgically by laparoscopic cholecystectomy followed by formal liver resection and lymphadenectomy, followed by adjuvant chemotherapy, which led to no evidence of disease (NED) at the last follow-up, while four patients were treated by palliative chemotherapy, one of them had ERCP with stenosis. Finally, the remaining five patients received BSC. Mortality in stage III patients was 63.6%. 

Tumour grading

Ten tumours (21.7%) were graded as grade one tumours (well-differentiated); seven patients (15.2 %) had grade one adenocarcinomas (moderately differentiated), and 14 patients (30.4%) had grade three carcinomas (poorly differentiated). In contrast, 15 patients (32.6%) did not have pathological analysis due to a lack of biopsy, thus the unknown grade. There were no significant differences in the survival rates of the patients who had grade one, two, or three (p=0.82) carcinomas. 

Gallstones

Gallstones were present in 38 patients (82.6%) who were diagnosed with GBC. Of those 38 patients, 12 (31.6%) were treated surgically. Six out of those 12 patients had only a laparoscopic cholecystectomy, and the remaining six patients had a subsequent liver resection and lymphadenectomy. The remaining 26 patients (68.4%) who did not undergo surgery were palliated. Of those 26 patients, nine patients (34.6%) received palliative chemotherapy, and the remaining 17 patients (65.4%) had BSC. 

On the other hand, eight patients (17.3%) had no history of cholecystolithiasis. Of those, only one patient was treated by laparoscopic cholecystectomy for having a gallbladder polyp without any further treatment, with the intention of cure. Two patients had palliative chemotherapy, and the remaining six patients received BSC.

In those patients who had no gallstones, only one patient had a localised disease, without any nodal or distant metastasis (stages I/II), while the remaining seven patients were either stage III (three patients) or stage IV (five patients). Comparing these results, there was no significant difference between patients with cholelithiasis and those without regarding having an early-stage cancer (stage I/II) (*p*=0.05).

Patients with a history of cholecystolithiasis had a MOS of four months (CI 6.710 - 22.29 months), while those without a history of cholecystolithiasis had a shorter mean survival of two months (CI 0.760 to 16.440 months). However, this difference was not statistically significant (*p* =0.48).

## Discussion

GBC is a rare but aggressive malignancy with varying treatment approaches depending on the stage, patient condition, and tumour characteristics [23]. This study, conducted in a single centre in the UK, shows diagnostic discrepancy throughout the 10 years of the study, where zero cases were diagnosed in 2020 due to difficulties in accessing health care facilities during the COVID-19 pandemic. The study also focuses on the effect of those different modalities on the survival rates among patients with gallstones versus those without gallstones, and those who were included in the surgery group versus the palliative group. The study concluded that survival was better in the surgical group, where GBC was diagnosed incidentally following laparoscopic cholecystectomy for gallstones, and the tumour was discovered early. There was no difference in survival, however, between patients who had gallstones and those who didn’t.

Literature and current guidelines show that surgery remains the gold standard treatment for early-stage GBC. The type of surgery depends on the staging of the tumour and extent of invasion, where radical resection, including partial hepatectomy and lymphadenectomy, is recommended for T1b and T2. Moreover, T3 and T4 tumours should be treated with radical cholecystectomy. Nevertheless, whether the GBC is resectable or not forms a major surgical challenge [24]. Studies have shown that radical cholecystectomy has a positive effect on the overall survival (OS) and cancer-specific survival (CSS) in advanced stages [25,26]. In addition, a simple cholecystectomy might be enough for early-stage GBC (T1a), with five-year survival rates approaching 100% [6,27].

Moreover, chemotherapy plays a pivotal part in the treatment of GBC, particularly in advanced stages. Adjuvant chemotherapy is the best therapy for patients with advanced stages, i.e., stage III-IV GBC, after radical resection. Chemotherapy regimens such as gemcitabine and cisplatin have a very positive effect on OS [28]. Neoadjuvant chemotherapy, on the other hand, is increasingly used for locally advanced GBC (i.e., T3 tumours) to downstage tumours before surgery. However, its survival benefit in early-stage GBC (i.e., T1, T2) remains controversial [29]. For metastatic GBC, palliative chemotherapy is the standard of care, with combination regimens like gemcitabine and cisplatin showing improved OS compared to BSC [19]. In our study, 19 patients (40.4%) underwent chemotherapy. In 14 out of those 19 patients, chemotherapy was administered with palliative intent, with an 85.7% mortality rate, while only five patients received chemotherapy with curative intent following surgery, and the survival rate was 100% at the end of the follow-up period (median of 57 months).

BSC is reserved for patients with advanced disease who are not candidates for curative treatments, nor palliative chemoradiotherapy. BSC focuses on symptom relief, pain management, and improvement of the quality of life as well as end of life care. While BSC alone is associated with poor survival, it still plays a pivotal role in palliative care for patients with GBC [21].

Limitations of this study include being a retrospective study with some limitations to data extraction through electronic records. In addition, the tumour grade could not be analysed in 30% of the cases due to late diagnosis of cancer and fear of tumour contamination due to biopsy. As a result, survival analysis based on tumour grade was underpowered. Finally, the small sample size led to a low statistical power, posing a limitation to the generalisation of the study results.

## Conclusions

The management of GBC requires a multidisciplinary approach, with surgery and chemotherapy playing pivotal roles. Our study shows that incidentally discovered GBC after gallstone surgery carries better survival outcomes than symptomatically evident cancer. There was no difference in survival between patients with or without gallstones, which agrees with the current literature. Finally, gender differences did not pose survival differences in our studies, which differs from that of other studies. Radical surgery combined with adjuvant therapies offers the best survival outcomes for early-stage (TIb, T2) and locally advanced GBC (T3). Further research is needed to explore different modalities to screen, diagnose early, and effectively treat advanced cases of GBC, with better survival results.
